# Disruption of the mast cell carboxypeptidase A3 gene does not attenuate airway inflammation and hyperresponsiveness in two mouse models of asthma

**DOI:** 10.1371/journal.pone.0300668

**Published:** 2024-04-05

**Authors:** Ida Waern, Srinivas Akula, Venkata Sita Rama Raju Allam, Sowsan Taha, Thorsten B. Feyerabend, Magnus Åbrink, Sara Wernersson

**Affiliations:** 1 Department of Anatomy, Physiology and Biochemistry, Swedish University of Agricultural Sciences, Uppsala, Sweden; 2 Department of Medical Biochemistry and Microbiology, Uppsala University, Uppsala, Sweden; 3 Division for Cellular Immunology, German Cancer Research Center, Heidelberg, Germany; 4 Department of Biomedical Sciences and Veterinary Public Health, Swedish University of Agricultural Sciences, Uppsala, Sweden; Universidade Federal do Rio de Janeiro, BRAZIL

## Abstract

Mast cells are effector cells known to contribute to allergic airway disease. When activated, mast cells release a broad spectrum of inflammatory mediators, including the mast cell-specific protease carboxypeptidase A3 (CPA3). The expression of CPA3 in the airway epithelium and lumen of asthma patients has been associated with a Th2-driven airway inflammation. However, the role of CPA3 in asthma is unclear and therefore, the aim of this study was to investigate the impact of CPA3 for the development and severity of allergic airway inflammation using knockout mice with a deletion in the *Cpa3* gene. We used the ovalbumin (OVA)- and house-dust mite (HDM) induced murine asthma models, and monitored development of allergic airway inflammation. In the OVA model, mice were sensitized with OVA intraperitoneally at seven time points and challenged intranasally (i.n.) with OVA three times. HDM-treated mice were challenged i.n. twice weekly for three weeks. Both asthma protocols resulted in elevated airway hyperresponsiveness, increased number of eosinophils in bronchoalveolar lavage fluid, increased peribronchial mast cell degranulation, goblet cell hyperplasia, thickening of airway smooth muscle layer, increased expression of IL-33 and increased production of allergen-specific IgE in allergen-exposed mice as compared to mocktreated mice. However, increased number of peribronchial mast cells was only seen in the HDM asthma model. The asthma-like responses in *Cpa3*^-/-^ mice were similar as in wild type mice, regardless of the asthma protocol used. Our results demonstrated that the absence of a functional *Cpa3* gene had no effect on several symptoms of asthma in two different mouse models. This suggest that CPA3 is dispensable for development of allergic airway inflammation in acute models of asthma in mice.

## Introduction

Asthma is a chronic respiratory disease currently affecting around 300 million people in the world [1, 2]. The disease is associated with airway hyperresponsiveness (AHR) and inflammation leading to airway obstruction. The majority of asthma patients (50–70%) have a chronic type 2 inflammation in their airways, which is characterized by a T helper cell 2 (Th2) cytokine profile, IgE production, accumulation of eosinophils and activation of mast cells [3]. The type 2 asthmatic response can be triggered by environmental allergens, such as different tree pollens and house dust mite (HDM) sheddings [4].

It is widely recognized that mast cells are effector cells and play a central role for the pathophysiology in allergic diseases, such as allergic asthma. When activated, mast cells degranulate and release a number of pre-formed mediators from their secretory granules. These include proteases, bioactive amines, proteoglycans and cytokines [5, 6]. Mast cells also respond to various stimuli by de novo synthesis and release of additional biologically active mediators. Many of the mast cell mediators are acting proinflammatory, but mast cells also express mediators that can have anti-inflammatory and immunosuppressive functions, such as proteases that can deactivate proinflammatory cytokines [7]. The pre-formed and active mast cell-specific proteases, *i*.*e*. chymase, tryptase and carboxypeptidase A3 (CPA3), constitute up to 30% of the total content of the mast cell granules [8]. Due to their large volume fraction, the mast cell proteases most likely have a significant impact on the surrounding environment when released and could play an important role in the pathophysiology of asthma [9].

Among the mast cell proteases, tryptase and chymase have been studied more extensively in comparison to CPA3. Found in serum post degranulation, tryptase serves as a diagnostic marker for mast cell activation [9]. Tryptase has the ability to cleave a number of substrates and contribute to airway hyperresponsiveness in asthma models [7]. For example, deletion of the gene *Mcpt6*, which is coding for the tryptase mouse mast cell protease 6 (mMCP-6), has been shown to dampen the features of allergic airway inflammation, including BAL eosinophilia and AHR [10]. Consistent with similar studies, we demonstrated that nafamostat, a mast cell tryptase inhibitor, alleviated HDM-associated allergic airway inflammation [11]. Although mast cells are mainly associated with proinflammatory biological effects, the murine chymase mMCP-4 has been shown to have protective properties in experimental asthma, dampening AHR and eosinophilic inflammation, possibly by degrading IL-33, which is a cytokine driving type 2 inflammation [12, 13].

Less is known about the role of CPA3/*Cpa3* in asthma. Interestingly, several clinical studies have shown upregulation of mast cell CPA3 expression in asthmatic patients [14–16]. Based on immunohistological analyses, tryptase- and CPA3-positive mast cells have been shown to infiltrate the airway epithelia in asthma patients with a high Th2 cell count [14]. In sputum samples, increased gene expression of CPA3 has been associated with eosinophilia and prediction of corticosteroid response in asthma [16]. In addition, increased CPA3 expression has been associated with blood and airway eosinophilia in patients with chronic obstructive pulmonary disease [17]. Therefore, CPA3 appears to be a candidate biomarker and potential target in the treatment of type 2 asthmatic conditions.

We have previously reported that blockage of carboxypeptidase activity using the *Nerita versicolor* carboxypeptidase inhibitor (NvCI) dampened AHR and reduced goblet cell hyperplasia in a murine asthma model [18]. Hence, CPA3 or other members of the carboxypeptidase M14A subfamily, which are inhibited by NvCI [19], may contribute to symptoms in experimental asthma. To identify the individual role of CPA3 in this context, we tested the response in mice lacking the gene for CPA3 (*Cpa3*^-/-^), in two asthma models using ovalbumin (OVA) and house dust mite (HDM) allergens, respectively. Notably, the *Cpa3*^-/-^ mouse strain has a secondary protein deficiency in chymase 1 (CMA1), also called mouse mast cell protease 5 (mMCP-5) [20], presumably due to a strong interdependency between these proteases for proper storage in secretory granules [21]. Lung function measurements and inflammatory responses to both allergens were similar in wild type (WT) and *Cpa3*^-/-^ mice. Our results suggest that CPA3/*Cpa3* (and indirectly mMCP-5) does not have an effect on AHR or lung inflammation in the models tested.

## Materials and methods

### Mice

Mice with a targeted inactivation of the *Cpa3* gene (*Cpa3*^-/-^) and a concomitant deficiency in granule storage of mMCP-5 [20] on the C57BL/6 background were backcrossed for >12 generations to the BALB/c background. *Cpa3* deficiency was ascertained by routinely genotyping offspring from *Cpa3*^+/-^ breeding pairs. Genotyping was performed by PCR using combinations of three Primers (common 5’ oligo: GGA CTG TTC ATC CCC AGG AAC C; 3’-WT oligo: CTG GCG TGC TTT TCA TTC TGG; and 3’-KO oligo: GTC CGG ACA CGC TGA ACT TG), yielding 328 bp (*Cpa3* WT) and 200 bp (*Cpa3* KO) products (S1 Fig). Deletion of *Cpa3* was also confirmed by the absence of CPA activity in peritoneal cells from a *Cpa3*^-/-^ mouse (S1 Fig).

### Allergic airway inflammation

For OVA-induced allergic airway inflammation, we used a model omitting strong adjuvants that was initially described by Williams, C.M. et al. [22] and later used in our previous study [12]. Littermates of female and male WT or *Cpa3*^-/-^ BALB/c mice were immunized intraperitoneally (i.p.) with 10 μg of OVA (Sigma-Aldrich, St Louis, MO) in 150 μl PBS on days 1, 3, 6, 8, 10, 13 and 15. On days 31, 33 and 36 sensitized mice were anesthetized with isoflurane and challenged intranasally (i.n.) with 20 μg OVA in 20 μl PBS. Control mice were given PBS (without OVA) in the same volumes and at the same timepoints as given for OVA sensitization and challenge.

For HDM-induced airway inflammation, we used the same model as in our previous studies [13, 18] but with a new batch of HDM extract for which the optimal dose was first tested. Female and male *Cpa3*^-/-^ BALB/c mice or WT littermate controls were anesthetized with isoflurane and instilled i.n. with 25 μg HDM extract from the species *Dermatophagoides farinae* (Greer Laboratories, Lenoir, NC), dissolved in 30 μl PBS, on days 1, 4, 8, 11, 15 and 18. Control mice were given 30 μl PBS i.n. at the same timepoints. All animal experiments were approved by the local ethics committee in Uppsala (Uppsala djurförsöksetiska nämnd, Dnr 5.8.18-12873/2019) and were conducted in accordance with the EU Directive 2010/63/EU for animal experiments.

### Measurement of airway reactivity

Mice were anesthetized with pentobarbital sodium (50 mg/kg, Sigma-Aldrich), tracheostomized and intubated to a small animal ventilator (Buxco FinePointe, DSI, St. Paul, MN, USA). Mice were ventilated at 160 breaths/min with a tidal volume of 0.25 ml. Prior to measurements, mice were shortly ventilated to reach an acclimatization baseline. Lung function was assessed with a dose-response curve to increasing doses of aerosolized methacholine (Sigma-Aldrich) via the tracheal cannula. Lung resistance (RL) and dynamic compliance (Cdyn) are shown as % change from baseline.

### Bronchoalveolar lavage (BAL) and lung tissue sampling and homogenization

BAL fluid was collected as previously described [18]. Cytospin slides were prepared and the percent of leukocyte populations was determined after May Grünwald/Giemsa staining. For cytokine measurements, the right lung lobes were frozen on dry ice and stored at -80°C. Lung tissue was homogenized in PBS containing protease inhibitor cocktail (Roche Diagnostics, Mannheim, Germany) at a concentration of 50 mg lung tissue/ml buffer using a bead-based Precellys homogenizer (Precellys Evolution, Bertin Technologies, France). The homogenates were centrifuged at 800 x g at 4°C for 8 min, and supernatants were collected for cytokine measurements using ELISA (IL-4 from PeproTech, London, UK, and IL-33 from eBioscience, San Diego, CA) according to the manufacturer’s instructions.

### Lung histology

Lung lobes were preserved in 4% paraformaldehyde, paraffin-embedded, sectioned and stained with periodic acid-Schiff (PAS) for analysis of goblet cell hyperplasia. Slides were deparaffinized, hydrated, oxidized in 0.05% periodic acid for 5 min, washed in H_2_O, stained with Schiff’s reagent (Sigma-Aldrich) for 30 min, washed in H_2_O for 3 min and counterstained with Mayer’s hematoxylin for 2 minutes. Number of goblet cells per μm basement membrane (airway epithelia) was counted with a Nikon Microphot-FXA microscope (Bergström Instrument AB, Stockholm, Sweden) using a 40x objective lens and Eclipse Net software (version 1.20, Developed by Laboratory Imaging, Prague, Czech Republic). Thickness of the airway smooth muscle (ASM) layer around similar sized bronchi was measured on PAS-stained sections and the average thickness was calculated. For analyses of mast cells, lung tissue slides were stained with Toluidine blue stain as described previously [18]. Total numbers of mast cells around the larger bronchi on each lung tissue slide were counted. Degree of mast cell degranulation was given a score based on the number of degranulated mast cells per lung tissue slide as follows: no degranulated cell (score 0), 1 degranulated cell (score 1), 2–3 degranulated cells (score 2), and >3 degranulated cells (score 3). All lung tissue sections were analyzed blindly.

### Quantification of OVA- and HDM-specific IgE

The serum concentration of allergen-specific IgE in serum was determined using ELISA kits for IgE-anti-OVA and IgE-anti-HDM, respectively, according to the manufacturer’s instructions (Chondrex Inc., Woodinville, WA).

### Carboxypeptidase A-, trypsin-like- and chymotrypsin-like activity

Lung tissue was homogenized in PBS at a concentration of 50 mg lung tissue/ml buffer. The lung tissue homogenates were centrifuged at 800 x g at 4°C for 8 min, and supernatants were collected for further analysis. Peritoneal cells were collected from WT and *Cpa3*^-/-^ mice injected with 10 mL ice cold PBS. The cell suspension was centrifugated at 400 x g for 8 min, supernatant was discarded and the cell pellet was lysed in 1 mL PBS containing 5% Triton-X 100. Carboxypeptidase A (CPA) activity in lung tissue and peritoneal cells was measured using the chromogenic CPA substrate N-(4-Methoxyphenylazoformyl)-L-phenylalanine-OH, (M-2245/AAFP, Bachem, Bubendorf, Switzerland) according to the manufacturer’s instructions. The reaction was allowed to proceed for 3 h at room temperature and absorbance was measured at 405 nm using a FARCyte microplate reader (Amersham Biosciences, Uppsala, Sweden).

Trypsin-like and chymotrypsin-like activity was measured in lung tissue homogenates using the fluorogenic substrates Boc-Val-Pro-Arg-AMC (I-1120, Bachem) and Suc-Ala-Ala-Pro-Phe-AMC (I-1465, Bachem), according to manufacturer’s instructions. The reaction was allowed to proceed for 3 h in RT and fluorescence was measured at 360 nm (excitation) and 460 nm (emission) every 10 min using a FARCyte microplate reader (Amersham Biosciences).

### Statistical analysis

Statistical significances were calculated by two-way ANOVA for airway reactivity measurements and by one-way ANOVA (parametric or non-parametric) for differential cell counting, histology, enzyme activity and ELISA, using GraphPad Prism software version 10.2.0 (GraphPad Software Inc., San Diego, CA). All values are displayed as means ± SEM. p-values <0.05 were considered to be significant.

## Results

### Lung resistance and compliance in OVA asthma model

**Fig 1:**
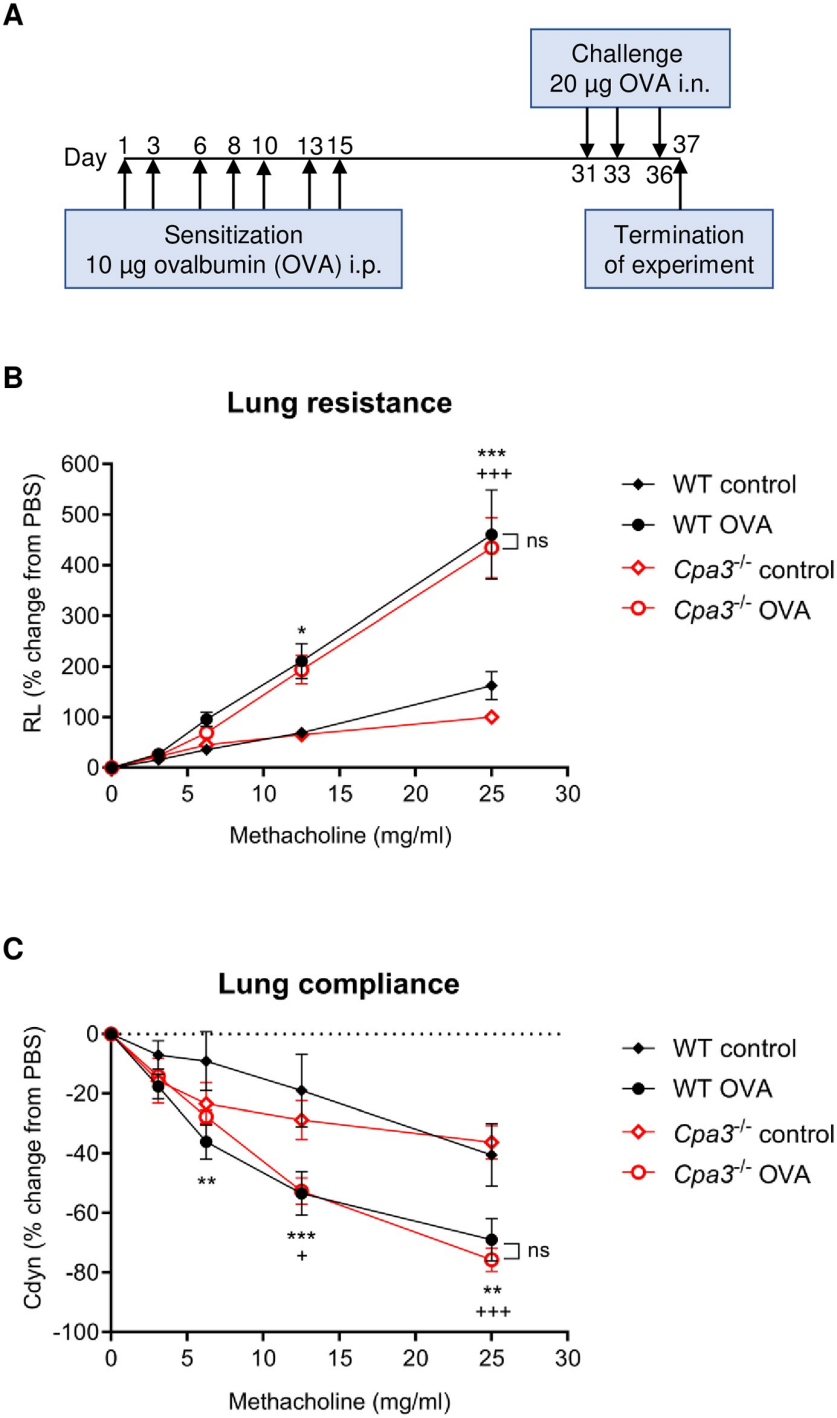
Lung resistance and dynamic compliance in ovalbumin (OVA)-induced asthma model. (**A**) Scheme of OVA-model. Wild type (WT) and *Cpa3*^-/-^ mice were i.p. immunized seven times with OVA followed by three i.n. instillations of OVA at the indicated days. Control mice were given PBS only at the same time points. (**B, C**) Lung resistance (R_L_) and dynamic compliance (C_dyn_) were measured with a Buxco FinePointe small animal ventilator. Results are expressed as mean ± SEM. WT OVA versus WT control: *p<0.05, **p<0.01 or ***p<0.001; *Cpa3*^-/-^ OVA versus *Cpa3*^-/-^ control ^+^*p*<0.05 or ^+++^*p*<0.001 (two-way ANOVA). n = 8–14 mice per group.

We first assessed the role of CPA3/*Cpa3* in an OVA-induced acute model of allergic asthma in which mice were sensitized and challenged with repeated doses of OVA according to the scheme in Fig 1A. Airway hyperresponsiveness (AHR) is a hallmark of asthma in humans and in animal models of asthma. To determine if AHR differed between WT and *Cpa3*^-/-^ mice during allergic airway inflammation, we used a Buxco ventilator to monitor lung resistance and compliance in response to increasing doses of aerosolized methacholine. We detected a significantly increased lung resistance in OVA-sensitized and challenged groups of mice compared to PBS controls (p<0.001, Fig 1B) and there was no significant difference between WT mice (WT OVA) and *Cpa3*^-/-^ mice (*Cpa3*^-/-^ OVA) in this respect. In line with the increased lung resistance, OVA-sensitized/challenged WT and *Cpa3*^-/-^ mice displayed a significant decrease in lung compliance as compared to their respective controls (p<0.001 and p<0.05 respectively, Fig 1C). In conclusion, the absence of *Cpa3* and indirect deletion of granule-stored mMCP-5 had no effect on AHR to methacholine in this model of allergic airway inflammation.

### BAL cell assessments in OVA model

**Fig 2:**
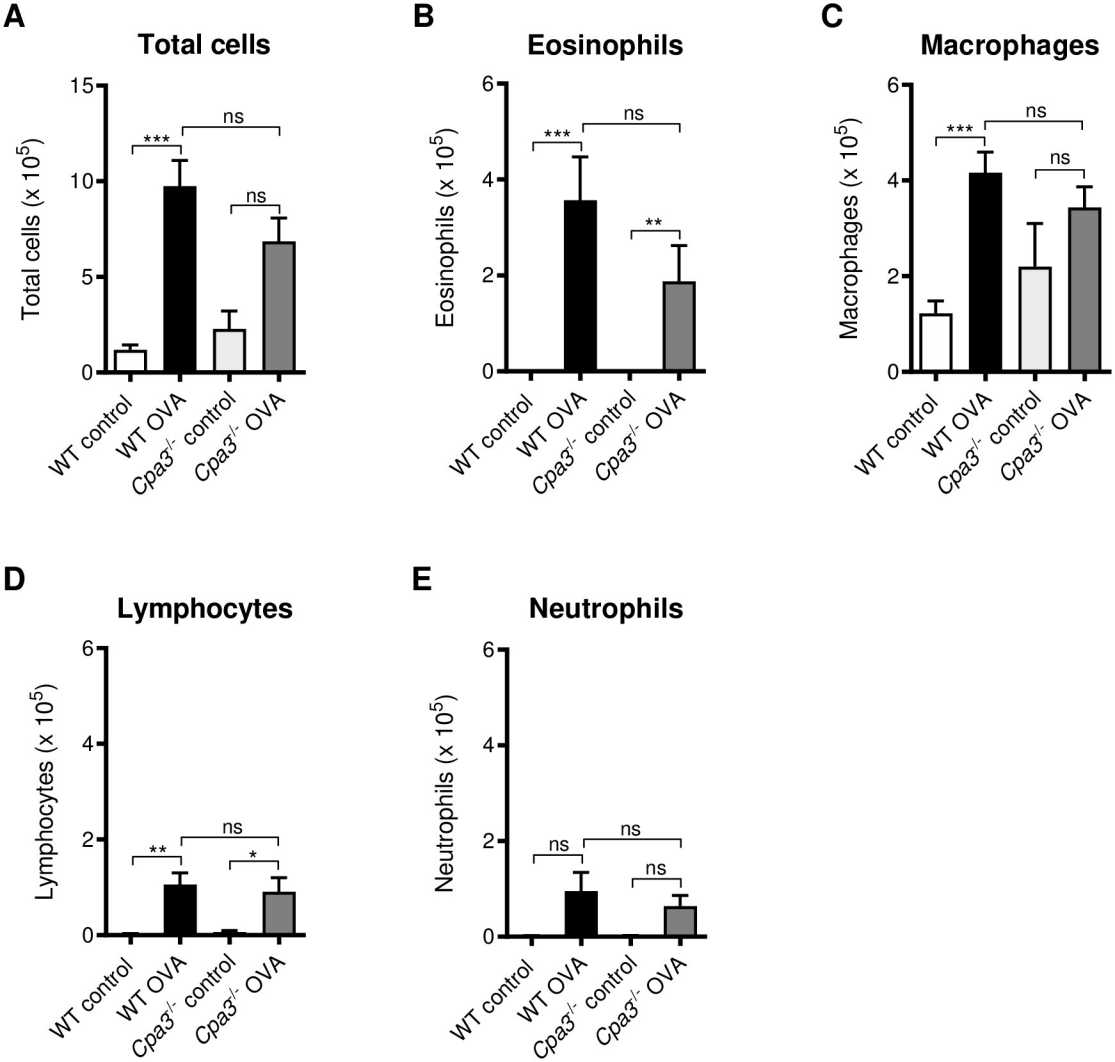
Leukocyte infiltration in bronchoalveolar lavage (BAL) following OVA challenge is not altered in *Cpa3*^-/-^ mice. Mice were i.p. immunized seven times with OVA followed by three i.n. instillations of OVA, as indicated in Fig 1. Control mice were given PBS only at the same time points. (**A-E**) Total cell and differential cell counts in BAL. Results are expressed as mean ± SEM. *p<0.05, **p<0.01, ***p<0.001 (one-way ANOVA, non-parametric). n = 8–13 mice per group.

To further investigate the role of CPA3/*Cpa3* in OVA-induced allergic airway inflammation the inflammatory cell infiltration in BAL was determined. OVA-sensitized/challenged WT but not *Cpa3*^-/-^ mice had significantly higher numbers of total BAL cells compared to controls (p<0.001, Fig 2A). The number of BAL cells in *Cpa3*^-/-^ OVA-treated mice did not differ significantly from that of WT OVA mice. As expected, the increase in total BAL cells in the OVA groups was mainly due to an increase in the number of eosinophils (p<0.001 and p<0.01, Fig 2B). There was a significant increase of macrophages in WT OVA mice (p<0.001) but not in *Cpa3*^-/-^ OVA mice (Fig 2C). Numbers of OVA-induced lymphocytes and neutrophils in BAL did not differ between *Cpa3*^-/-^ and WT mice (Fig 2D and 2E). In conclusion, there were no differences in the BAL cell types, comparing OVA-treated WT and *Cpa3*^-/-^ mice (Fig 2B–2E). These data suggest that CPA3 and mMCP-5 do not affect recruitment of inflammatory cells to the BAL following OVA sensitization and challenge.

### Mast cell numbers and degranulation in OVA model

**Fig 3:**
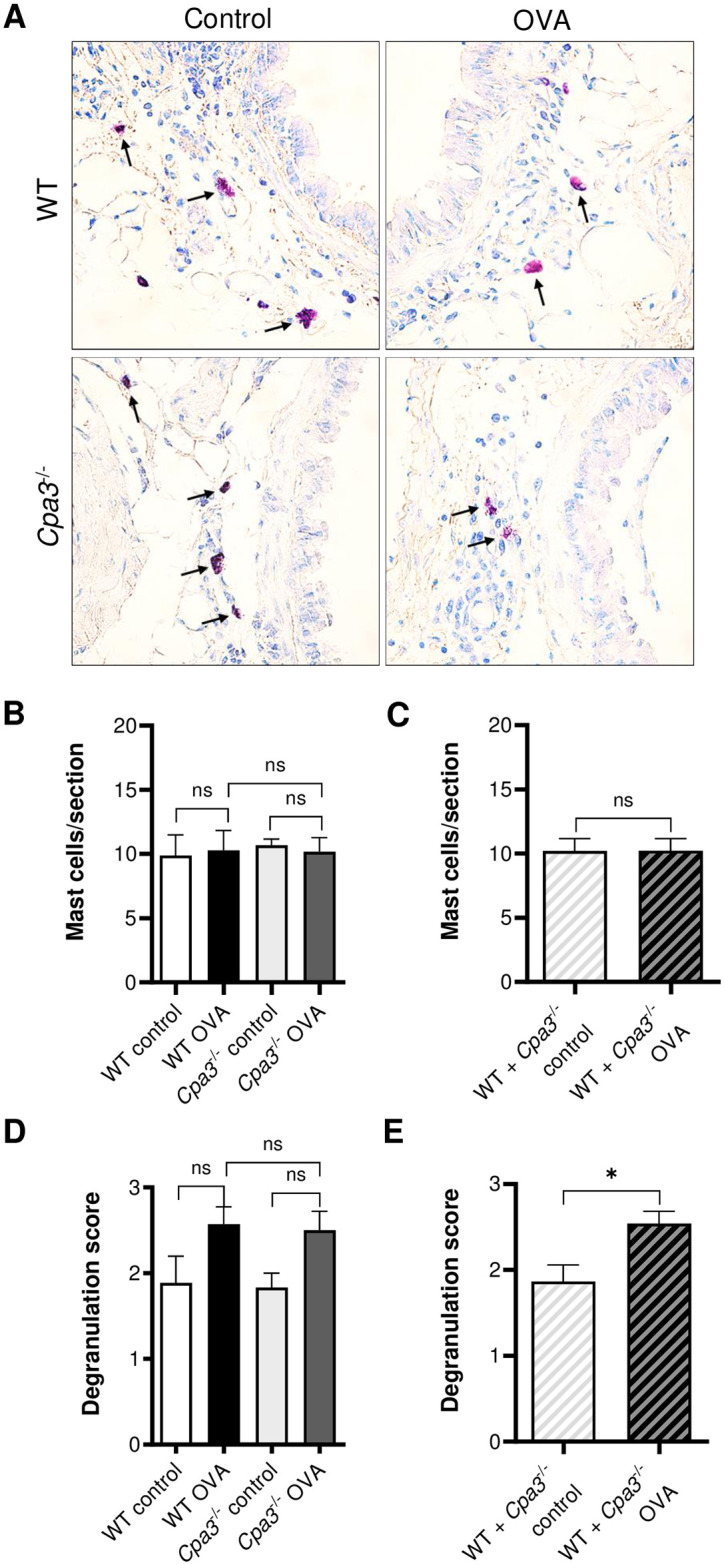
OVA-induced mast cell degranulation in WT and *Cpa3*^-/-^ mice. Mice were i.p. immunized seven times with OVA followed by three i.n. instillations of OVA, as indicated in Fig 1. Control mice were given PBS only at the same time points. (**A**) Mast cells (arrows) near the primary bronchi on lung sections stained with Toluidine blue. (**B**) Number of peribronchial mast cells per lung section (n = 6–9). (**C**) Number of peribronchial mast cells per lung section in pooled WT + *Cpa3*^-/-^ data (n = 13–15). (**D**) Degranulation score of peribronchial mast cells (n = 6–9). (**E**) Degranulation score of peribronchial mast cells in pooled WT + *Cpa3*^-/-^ data (n = 13–15). All lung tissue sections were blindly scored/measured. Results are expressed as mean ± SEM. *p<0.05 (one-way ANOVA, non-parametric).

To assess the effect of OVA-treatment on mast cell numbers and degranulation, histological examination was performed on lung tissue sections stained with Toluidine blue. In accordance with previous studies, lung mast cells were almost exclusively found around the larger primary bronchi and very rarely seen in the alveolar parenchyma. Numbers of mast cells around the large bronchi were similar between all groups of mice and did not increase in response to OVA treatment (Fig 3A–3C). A non-significant trend of OVA-induced degranulation of mast cells was seen for WT and *Cpa3*^-/-^ mice when all four groups were analyzed with non-parametric one-way ANOVA (p = 0.0805). Since no difference was seen in degranulation between WT and *Cpa3*^-/-^ mice, we analyzed pooled data (WT + *Cpa3*^-/-^) and found a higher mast cell degranulation score in the OVA group than in controls (p<0.05, Fig 3E). These findings suggests that mast cell degranulation, but not mast cell numbers, is increased in this OVA model.

### Goblet cell hyperplasia and airway smooth muscle thickness in OVA model

**Fig 4:**
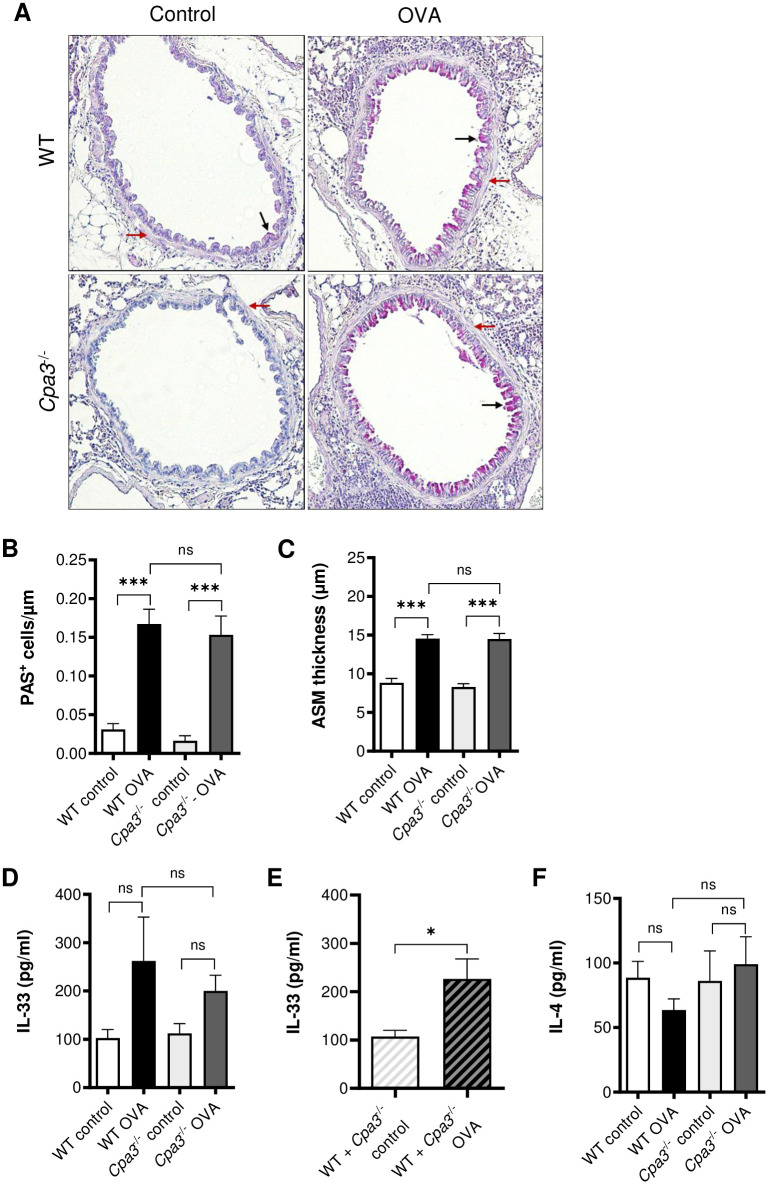
OVA-induced tissue inflammation and remodelling in WT and *Cpa3*^-/-^ mice. Mice were i.p. immunized seven times with OVA followed by three i.n. instillations of OVA, as indicated in Fig 1. Control mice were given PBS only at the same time points. (**A**) Histology of lung sections stained with periodic acid-Schiff’s (PAS) stain. Goblet cells (black arrows) and airway smooth muscle layer (red arrows) around larger bronchi. (**B**) Number of PAS^+^ cells per μm airway epithelia. (**C**) Thickness of the airway smooth muscle (ASM) layer around similar sized bronchi was measured and the average thickness was calculated. All lung tissue sections were blindly scored/measured. (**D, E**) Concentrations of IL-33 and IL-4 in lung homogenates were measured with ELISA. Results are expressed as mean ± SEM. ***p<0.001 (one-way ANOVA). n = 6–13 mice per group.

Next, we analyzed lung tissue sections for the effects of *Cpa3* deficiency on goblet cell hyperplasia and the airway smooth muscle (ASM) thickness. OVA sensitized and challenged mice demonstrated a marked increase in the number of PAS-positive cells compared to controls (p<0.001, Fig 4A and 4B). In accordance with the BAL data, there was no difference in goblet cell numbers between OVA-treated WT and *Cpa3*^-/-^ mice (Fig 4B). Similar results were observed for the ASM thickness, i.e. no baseline difference was seen when comparing WT and *Cpa3*^-/-^ control mice and a profound increase in the ASM thickness was observed in OVA-treated WT and *Cpa3*^-/-^ mice compared to controls (p<0.001, Fig 4C). However, there was no difference in the thickening of the ASM layer comparing OVA-treated *Cpa3*^-/-^ and WT mice (Fig 4C).

### Cytokine expression in lung tissue in OVA model

IL-33 and IL-4 are two cytokines associated with type 2 inflammation. Both cytokines can be produced by mast cells but in mouse lungs IL-33 is mainly produced by type-2 pneumocytes [23] and IL-4 could also be produced by Th2 cells. Lung tissue homogenates were analyzed for IL-33 and IL-4, using ELISA (Fig 4D–4F). There were no statistically significant differences in the cytokine levels between WT and *Cpa3*^-/-^ mice or between their respective OVA-treated and control groups (Fig 4D and 4F). However, a significant increase in IL-33 was seen in all OVA-treated mice when analyzing pooled data for WT and *Cpa3*^-/-^ mice (Fig 4E).

### OVA-specific IgE in OVA model

**Fig 5:**
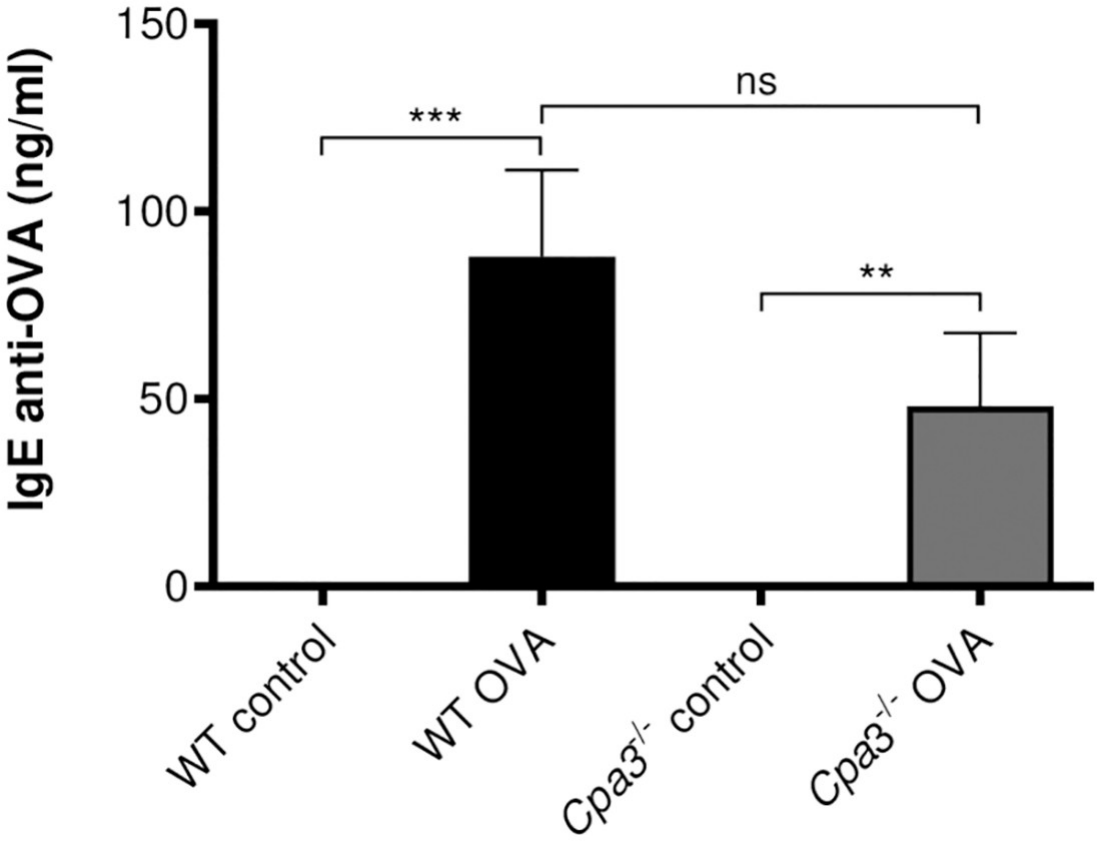
Increased OVA-specific IgE in OVA-treated WT and *Cpa3*^-/-^ mice. Mice were i.p. immunized seven times with OVA followed by three i.n. instillations of OVA. Control mice were given PBS at the same time points. Concentration of OVA-specific IgE was measured in serum using ELISA. Results are expressed as mean ± SEM. **p<0.01 or ***p<0.001 (one-way ANOVA, non-parametric). n = 8–13 mice per group.

Increased levels of allergen-specific IgE is a characteristic feature of allergic asthma. The OVA-specific IgE was increased in OVA-treated WT and *Cpa3*^-/-^ mice compared to controls (Fig 5). In agreement with the lung function, BAL cell and histology data, there were similar levels of OVA-specific IgE comparing the OVA-treated WT and *Cpa3*^-/-^ groups.

### Lung resistance and compliance in HDM model

**Fig 6:**
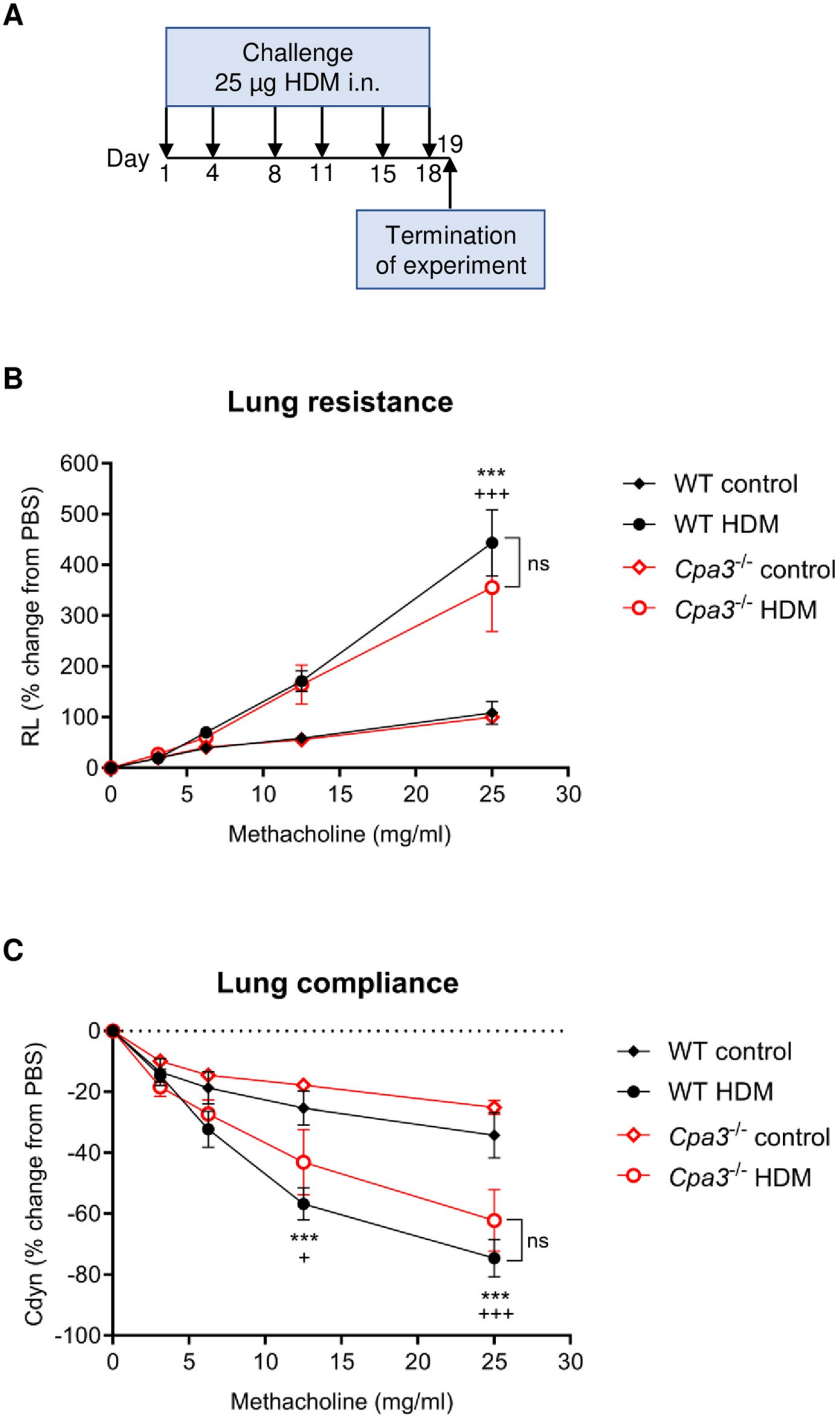
Lung resistance and dynamic compliance in house dust mite (HDM)-induced asthma model. (**A**) Scheme of the HDM model. Mice received either HDM or PBS (controls) twice a week for 3 weeks at the indicated days. (**B, C**) Lung resistance (R_L_) and dynamic compliance (C_dyn_) were measured with a Buxco FinePointe ventilator. Results are expressed as mean ± SEM. WT HDM versus WT control: ***p<0.001; *Cpa3*^-/-^ HDM versus *Cpa3*^-/-^control ^+^*p*<0.05, ^+++^*p*<0.001 (two-way ANOVA). n = 8–13 mice per group.

HDM is a physiologically relevant allergen known to cause asthma in humans and the allergen is also used in mouse models of allergic airway inflammation. In order to investigate the role of CPA3 and mMCP-5 in HDM-induced allergic asthma, WT and *Cpa3*^-/-^ mice were given HDM intranasally (i.n.) twice weekly for three weeks (Fig 6A). Similar to the OVA-model, mice challenged with HDM exhibited AHR as demonstrated by an increased lung resistance and decreased compliance compared to control mice, however the lung resistance and compliance were unaffected by the *Cpa3* deficiency (Fig 6B and 6C).

### BAL cell assessments in HDM model

**Fig 7:**
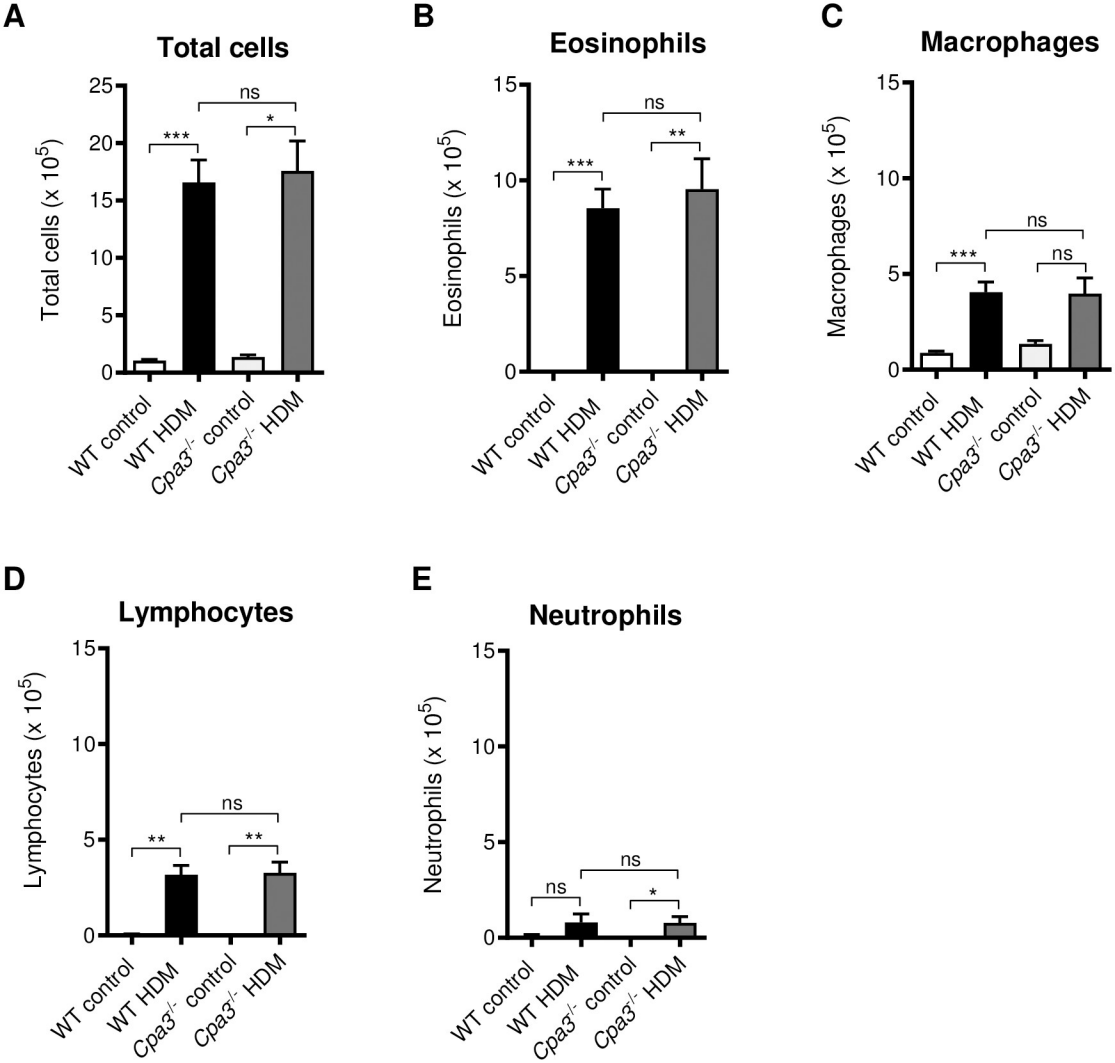
Inflammatory cells in BAL post HDM challenge is not altered in *Cpa3*^-/-^ mice. Mice received either HDM or PBS (controls) twice a week for 3 weeks. (**A**) Total cells, (**B**) eosinophils, (**C**) macrophages, (**D**) lymphocytes and (**E**) neutrophils were measured in the BAL fluid. Results are expressed as mean ± SEM. *p<0.05, **p<0.01 or ***p<0.001 (one-way ANOVA, non-parametric). n = 5–12 mice per group.

Analysis of the cells in BAL confirmed that treatment with HDM resulted in a dramatic increase in the number of inflammatory cells in BAL (Fig 7A) mainly due to an increased number of eosinophils (p<0.001 and p<0.01, Fig 7B). Similar to the OVA-model, HDM-treated *Cpa3*^-/-^ mice showed no difference in the total number of cells or numbers of eosinophils in BAL compared to WT mice. In addition, in HDM-treated WT mice there was an increased number of macrophages and lymphocytes compared to WT controls (p<0.001 and p<0.01 respectively, Fig 7C and 7D). Numbers of lymphocytes and neutrophils in BAL was increased in HDM-treated *Cpa3*^-/-^ mice compared to controls (p<0.001 and p<0.05, Fig 7D and 7E). However, the macrophage number was not significantly increased in HDM-treated *Cpa3*^-/-^ mice compared to control (Fig 7C). In agreement with the OVA-model, no differences were seen in the cell types investigated in BAL, comparing HDM-treated WT and *Cpa3*^-/-^ mice (Fig 7B–7E).

### Mast cell numbers, degranulation and protease activity in HDM model

**Fig 8:**
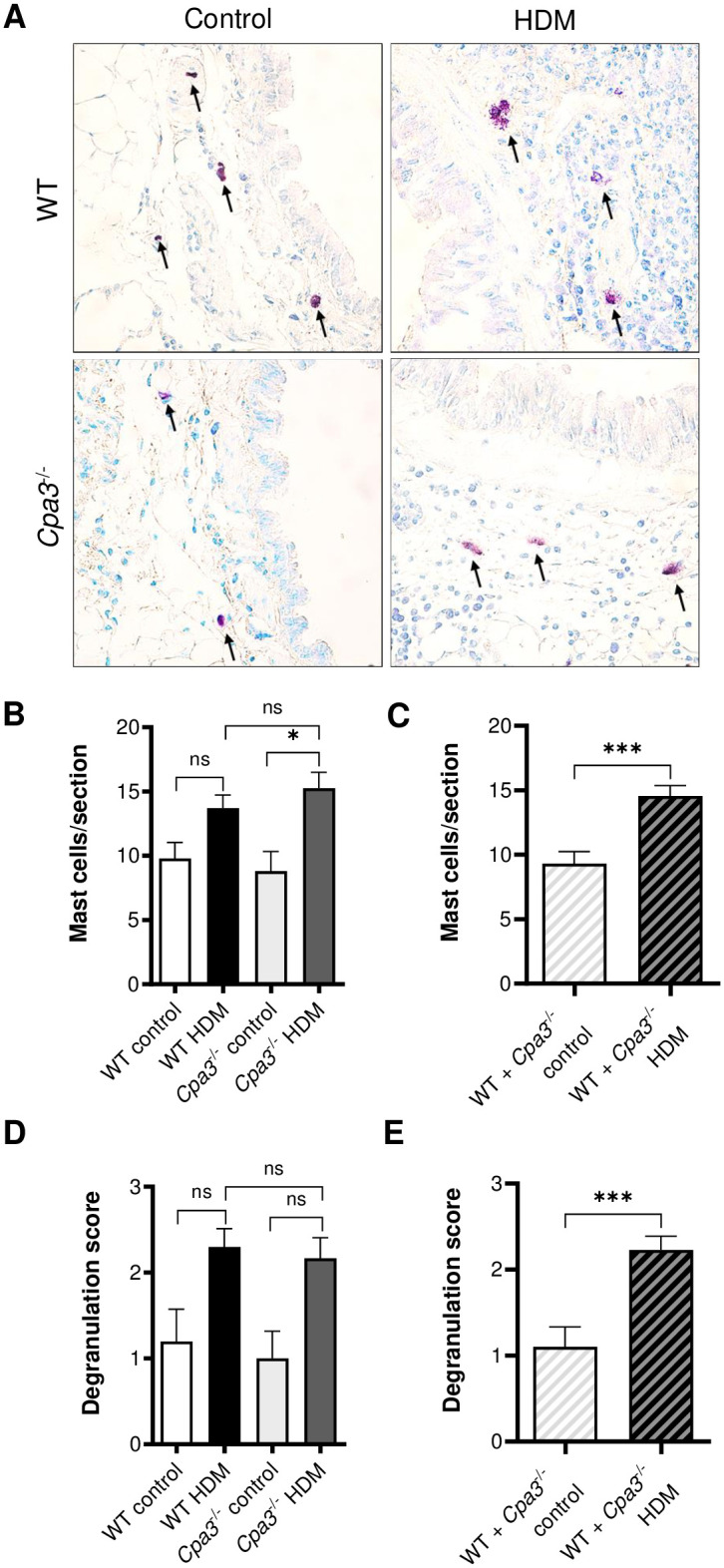
HDM-induced mast cell accumulation and degranulation in WT and *Cpa3*^-/-^ mice. Mice received either HDM or PBS (controls) twice a week for 3 weeks. (**A**) Mast cells (arrows) near the primary bronchi on lung sections stained with Toluidine blue. (**B**) Number of peribronchial mast cells per lung section (n = 5–12). (**C**) Number of peribronchial mast cells per lung section in pooled WT + *Cpa3*^-/-^ data (n = 10–22). (**D**) Degranulation score of peribronchial mast cells (n = 5–12). (**E**) Degranulation score of peribronchial mast cells in pooled WT + *Cpa3*^-/-^ data (n = 10–22). All lung tissue sections were blindly scored/measured. Results are expressed as mean ± SEM. *p<0.05 or ***p<0.001 (one-way ANOVA, non-parametric).

We analyzed the accumulation and degranulation of mast cells in the HDM model by histological examination of lung tissue sections (Fig 8A). In contrast to the OVA model, there was an increased number of mast cells around the large bronchi in the HDM model. This was demonstrated by the higher number of mast cells in HDM-treated *Cpa3*^-/-^ mice compared to controls (p<0.05, Fig 8B). In addition, there was a higher number of mast cells in all HDM-treated mice compared to all controls, when analyzing pooled data for WT and *Cpa3*^-/-^ mice (p<0.001, Fig 8C). Similar to the OVA model, there was a trend of increased mast cell degranulation in response to HDM in both WT and *Cpa3*^-/-^ mice and (Fig 8D), and we found a significantly increased degranulation when analyzing the combined response of all HDM-treated mice (p<0.001, Fig 8E). These findings suggest that the HDM model can induce both accumulation and degranulation of mast cells in the lung tissue around the large bronchi. This notion was further supported by the increased protease activity related to mast cell tryptase (trypsin-like activity) and chymase (chymotrypsin-like activity) in the lung tissue of HDM-treated mice, with no differences seen between WT and *Cpa3*^-/-^ mice (S2 Fig). These measurements were performed by using highly sensitive fluorogenic substrates. CPA3 activity may be measured by using a CPA-specific substrate (M-2245/AAFP). Due to chromogenic labeling, this substrate is less sensitive for measurements in tissues and we obtained values below the detection level in mouse lung tissue in the present study (S1 Fig). However, the presence of CPA3 protein in resident lung mast cells in mice has been confirmed in previous studies [12, 24]. Hence, the release of CPA3 around the larger bronchi in the HDM model is indirectly supported by our data showing an accumulation of mast cells, increased mast cell protease activity and increased mast cell degranulation in lungs of HDM-treated mice.

### Goblet cell hyperplasia and ASM thickness in the HDM model

**Fig 9:**
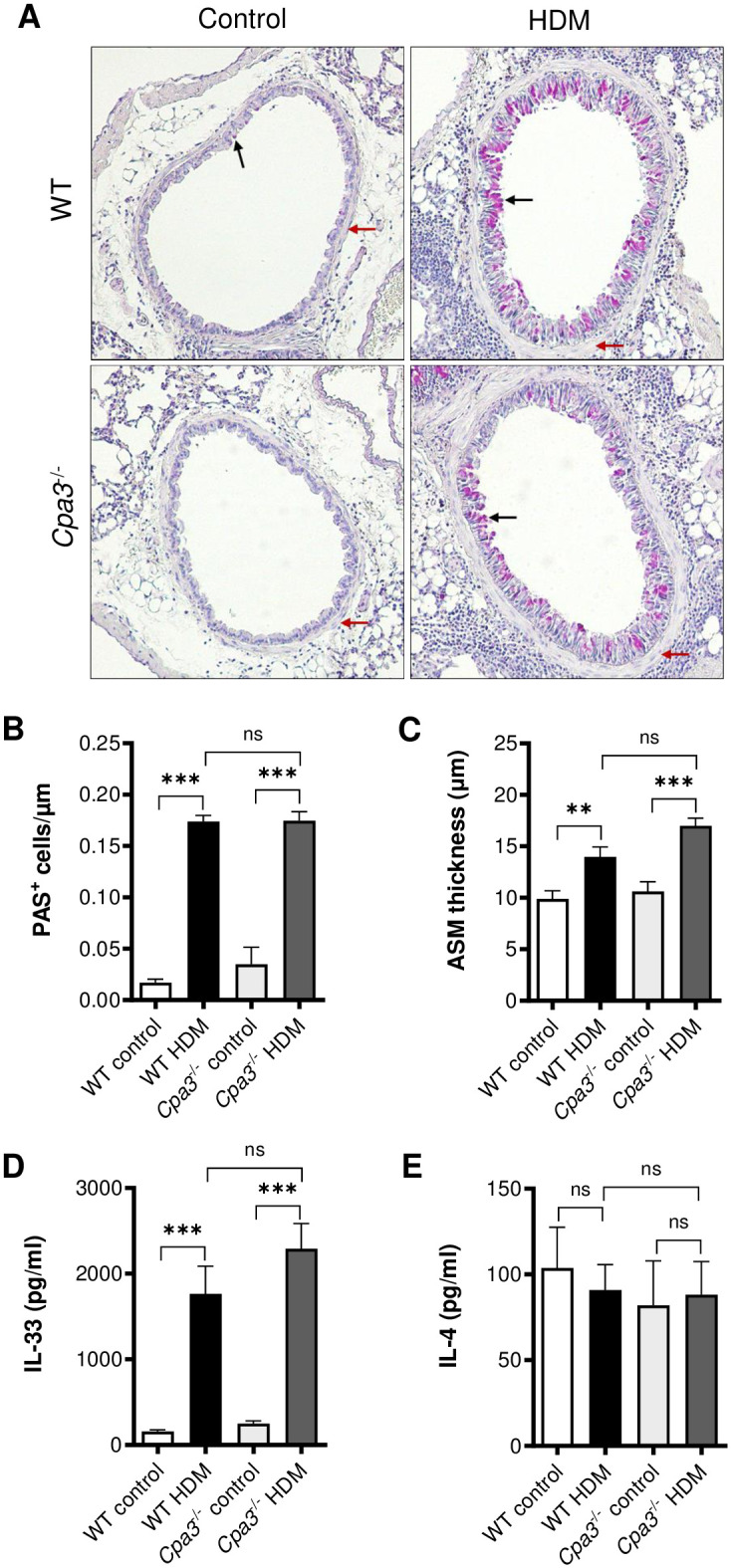
Increased tissue remodelling and lung tissue IL-33 in HDM-treated WT and *Cpa3*^-/-^ mice. WT and *Cpa3*^-/-^ mice received either HDM or PBS (controls) twice a week for 3 weeks. (**A**) Histology of lung sections stained with periodic acid-Schiff’s (PAS) stain. Goblet cells (black arrows) and airway smooth muscle layer (red arrows) around larger bronchi. (**B**) Goblet cell hyperplasia measured by number of PAS^+^ cells per μm airway epithelia. (**C**) Thickness of the airway smooth muscle (ASM) layer around similar sized bronchi was measured and the average thickness was calculated. All lung tissue sections were blindly scored/measured. (**D, E**) Concentrations of IL-33 and IL-4 in lung homogenates were measured with ELISA. Results are expressed as mean ± SEM. **p<0.01 or ***p<0.001 (one-way ANOVA). n = 7–21 mice per group.

Lung tissue sections were prepared and stained with PAS to assess goblet cell hyperplasia (Fig 9A). Examination of the stained sections demonstrated a marked increase in the number of PAS-positive cells in HDM-treated groups compared to controls (p<0.001 and p<0.01, Fig 9B) with no difference when comparing HDM-treated WT and *Cpa3*^-/-^ mice (Fig 9B). Next, we analyzed the effects of HDM-treatment and *Cpa3* deficiency on the ASM layer (Fig 9C). HDM-treatment caused an increased thickening of the ASM layer compared to control mice (p<0.05 and p<0.001, Fig 9C), however, no difference in the thickness was detected when comparing HDM-treated WT and *Cpa3*^-/-^ groups (Fig 9C).

### Cytokine expression in lung tissue in HDM model

We previously reported that IL-33 levels in the lung tissue were increased after HDM exposure [13]. In agreement with our earlier publication, HDM-treatment resulted in increased IL-33 levels in the lung (p<0.001 Fig 9D). However, no significant difference in the IL-33 levels between HDM-treated WT and *Cpa3*^-/-^ mice was detected. Levels of the Th2 cytokine IL-4 in homogenized lung tissues were unaltered in this model (Fig 9E).

### HDM-specific IgE in HDM model

**Fig 10:**
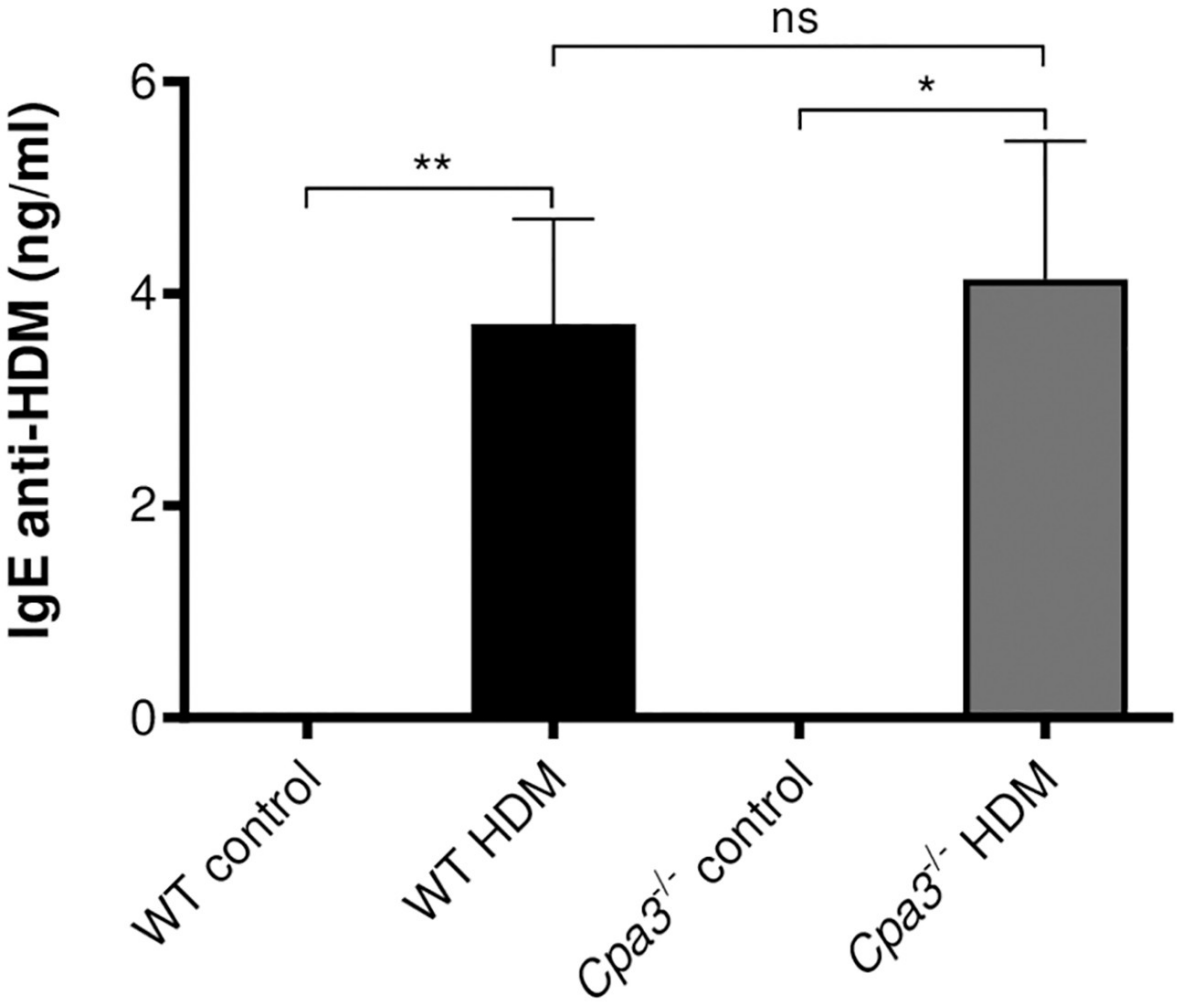
Increased HDM-specific IgE in HDM-treated WT and *Cpa3*^-/-^ mice. WT and ***Cpa3***^**-/-**^ mice received either HDM or PBS (controls) twice a week for 3 weeks. IgE specific for HDM was measured using ELISA. Results are expressed as mean ± SEM. *p<0.05 or **p<0.01 (1-way ANOVA, non-parametric). n = 7–19 mice per group.

To assess the effect of *Cpa3* deficiency on allergic sensitization to HDM, we measured HDM-specific IgE in serum. The serum levels of HDM-specific IgE were increased in HDM-treated groups compared to controls (Fig 10). We did not detect any differences in HDM-specific IgE between WT and *Cpa3*^-/-^ mice.

## Discussion

In this study, we sought to determine the role of mast cell protease CPA3 in allergic airway inflammation. To this end, we studied the development of allergic airway inflammation in a mouse strain genetically lacking *Cpa3* and concomitantly lacking granule-stored mMCP-5, using two different experimental protocols, the OVA- and HDM-induced asthma models. Both models induced a significant increase in airway resistance, inflammatory cells in BAL (particularly eosinophils), peribronchial mast cell degranulation, mucus-producing cells and airway smooth muscle thickness. However, a greater level of inflammation was seen when using the HDM- as compared to the OVA-induced protocol, and increased mast cell numbers were only seen in the HDM-model. Differences between models could be due to the stronger adjuvant effect elicited by components of the HDM whole extract compared to the purified OVA preparation. Taken together, we here demonstrated that deletion of *Cpa3* did not have an impact on AHR, inflammatory cell infiltration in BAL, peribronchial mast cell number and degranulation, cytokines in lung tissue, goblet cell hyperplasia, smooth muscle remodeling or serum levels of specific IgE in two models of asthma. These findings suggest that CPA3/*Cpa3* and mMCP-5 are dispensable for the development of several key features of allergic asthma, both in a milder asthma protocol, using OVA, and in a stronger and more physiologically relevant asthma protocol, using HDM as the allergen.

Although *Cpa3* and mMCP-5 deficiency in mice did not affect symptoms in acute asthma models in the present study, we cannot exclude a role for CPA3 in human chronic airway diseases such as asthma and chronic obstructive pulmonary disease (COPD). Findings in line with the involvement of CPA3 in chronic airway diseases comes from a number of studies demonstrating an increased CPA3 protein or gene expression in sputum or lung tissue from patients with asthma or COPD [15, 17, 25]. However, there is limited information about the potential role of CPA3 in the disease mechanisms in asthma. Several target proteins or peptides may be cleaved by CPA3 during allergic airway inflammation, including the substrates identified thus far for CPA3, i.e. endothelin-1, angiotensin I, neurotensin, and apolipoprotein B [9]. In addition, it has been shown that elevated CPA3 mRNA expression is associated with pathogenic remodeling of the extracellular matrix in COPD lungs [26]. This suggests that CPA3 could have both important protective functions, e.g. by deactivating pro-inflammatory cytokines, as well as pathogenic tissue remodeling properties.

The OVA and HDM-models have previously been useful to identify molecular mechanisms underlying disease pathogenesis. For example, using these two models, we found augmented responses in mice lacking the gene for the mast cell chymase mMCP-4 (*Mcpt4*), hence demonstrating that mast-cell related mechanisms can be identified in murine asthma models [12, 13]. We have previously shown that substantial amounts of CPA3 can be detected in mouse lungs using immunoblot [12], and others have demonstrated the presence of CPA3 in lung mast cells of BALB/c mice using immunohistochemistry [24]. Hence, the increased mast cell degranulation demonstrated in both OVA- and HDM-induced models, implies that CPA3 could be released in response to allergen exposure in mouse models and could potentially affect the inflammation by cleaving various substrates. However, the lack of altered response in *Cpa3*^-/-^ mice in the two models of asthma suggests that the released CPA3 does not substantially contribute to, or reduce, any of the major symptoms related to asthma.

We have previously shown that blockage of carboxypeptidase activity, using Nerita versicolor carboxypeptidase inhibitor (NvCI), could suppress AHR in a mouse model of HDM-induced asthma [18]. NvCI is a strong natural inhibitor of the carboxypeptidase M14A subfamily, which among others includes the mast cell CPA3, the digestive enzymes CPA1, CPA2, and CPB1, and the plasma enzyme CPB2, also called thrombin activatable fibrinolysis inhibitor [19]. Although CPA3 was a strong candidate for the protective effect by NvCI seen in experimental asthma [18], this notion was not supported by the intact asthma-like response seen in CPA3-deleted mice in the present study. Together, these findings suggest that members of the M14A subfamily, other than CPA3, could potentially contribute to features of experimental asthma. However, in order to discriminate between the role of different M14A carboxypeptidases in asthma, future studies including selective inhibitors are warranted. It should be noted that compared to our previous studies in HDM models, we here used a new batch of HDM that required a higher dose to induce similar responses. Direct comparisons between studies should therefore be done with caution.

In mice, CPA3 and the chymase mMCP-5 are dependent on each other for being stored in the mast cell granules [20]. For example, mast cells in *Cpa3*^-/-^ mice will also lack granule stored mMCP-5 protein despite normal *Mcpt5* mRNA expression [27]. Although mMCP-5 could potentially be secreted directly without pre-storage in granules, it can be assumed that CPA3 deletion will severely reduce the amounts of mMCP-5 released in the lungs of asthmatic mice. A decreased mMCP-5 level in the *Cpa3*^-/-^ mice could have influenced the response in the asthma models. However, we did not find any major effects on the asthma response in *Cpa3*^-/-^ mice, suggesting that both CPA3 expression and mMCP-5 granule storage are dispensable for development of an asthma response in experimental models. Moreover, it seems unlikely that an impairment of mMCP-5 storage could have masked any potential effects on the asthma model caused by the lack of CPA3 *per se*, although this possibility cannot be excluded.

Mast cells are known to initiate a rapid response to various stimuli, including allergens, and it is widely recognized that mast cells contribute to the manifestations of asthma by the release of pro-inflammatory cytokines, proteases and lipid mediators [5]. Intriguingly, mast cell mediators can also exhibit anti-inflammatory and protective properties, which illustrates the heterogeneous nature of mast cell functions. For example, the mast cell tryptase has been shown to mainly be pro-inflammatory in mouse models of asthma, contributing to airway hyperresponsiveness although tryptase also has the ability to degrade pro-inflammatory cytokines [10, 28, 29]. Similarly, mast cell chymase may exhibit both pro- and anti-inflammatory effects in the context of airway inflammation, as it has been shown that chymase can promote fibrosis by degradation of fibronectin and collagen [30] but can also reduce AHR [12, 13] and degrade various type 2 cytokines and chemokines [31, 32]. In contrast to tryptase and chymase, there is limited information regarding the possible roles of CPA3 in asthma. Given the identified targets for CPA3 in combination with the increased expression of CPA3 in asthmatic patients, we hypothesized that CPA3 could affect the inflammatory response and promote tissue remodeling in the asthmatic lung. The present study, using *Cpa3*^-/-^ mice, demonstrated that CPA3 is dispensable for development of several key features of experimental asthma, including AHR, inflammatory cell accumulation, airway remodeling and allergic sensitization. Hence, these findings do not support a crucial detrimental or protective role of CPA3 in asthma.

## Supporting information

S1 Fig Genotyping and CPA activity. (PDF)

S2 Fig Trypsin-like and chymotrypsin-like activity. (PDF)

S1 File Data of the study. (XLSX)

## Abbreviations

AHR: airway hyperresponsiveness
ASM: airway smooth muscle
BAL: bronchoalveolar lavage
CPA3/Cpa3: carboxypeptidase A3
HDM: house dust mite
i.n: intranasal
i.p: intraperitoneal
mMCP/Mcpt: mouse mast cell protease
OVA: ovalbumin
PAS: periodic acid-Schiff
Th2: T-helper cell type 2
WT: wild type
