# Supplementary material for: Disruption of the mast cell carboxypeptidase A3 gene does not attenuate airway inflammation and hyperresponsiveness in two mouse models of asthma
Source: PLoS One. 2024 Apr 5;19(4):e0300668. doi: 10.1371/journal.pone.0300668 (PMC10997103; doi:10.1371/journal.pone.0300668)
Supplement: S1 Fig — (PDF) [file pone.0300668.s001.pdf]

Supplementary Fig 1.

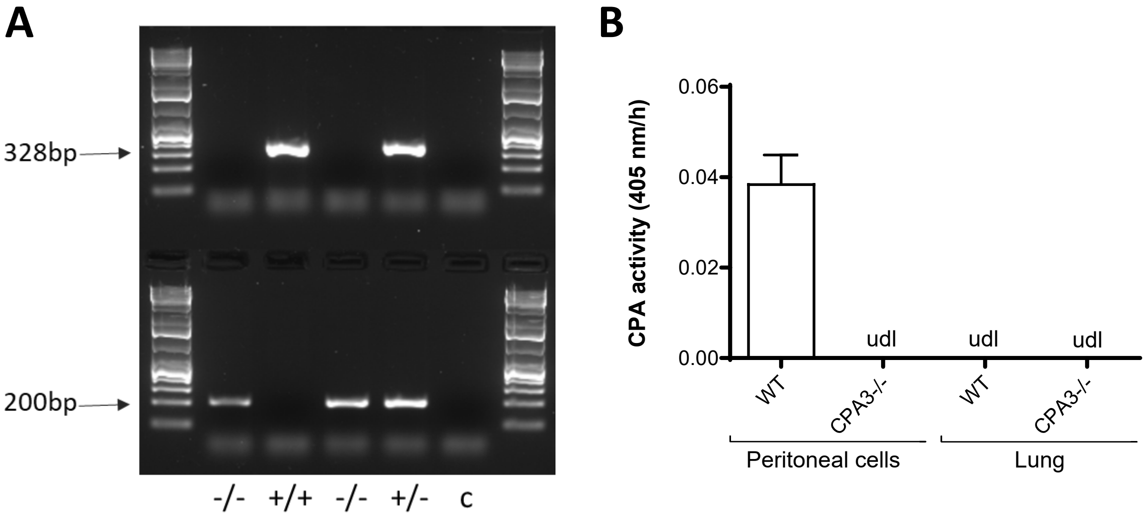

Figure S1. Genotyping and measurements of CPA activity in *Cpa3*$^{-/-}$ mice. (**A**) Mice were tested by PCR for presence of the *Cpa3* wildtype (upper gel) or *Cpa3* Knockout (lower gel) alleles. Knockout mice (-/-) only yield the 200 bp fragment in the lower PCR, wildtype mice (+/+) only yield the 328 bp fragment in the upper PCR and heterozygous mice (+/-) show both bands. ”c” is water control.

(**B**) Peritoneal cells and lung tissue homogenates from WT and *Cpa3*$^{-/-}$ mice (n=2) were assayed for CPA activity using the chromogenic substrate M-2245. udl, under detection level.
