# Supplementary material for: Disruption of the mast cell carboxypeptidase A3 gene does not attenuate airway inflammation and hyperresponsiveness in two mouse models of asthma
Source: PLoS One. 2024 Apr 5;19(4):e0300668. doi: 10.1371/journal.pone.0300668 (PMC10997103; doi:10.1371/journal.pone.0300668)
Supplement: S2 Fig — (PDF) [file pone.0300668.s002.pdf]

Supplementary Fig 2.

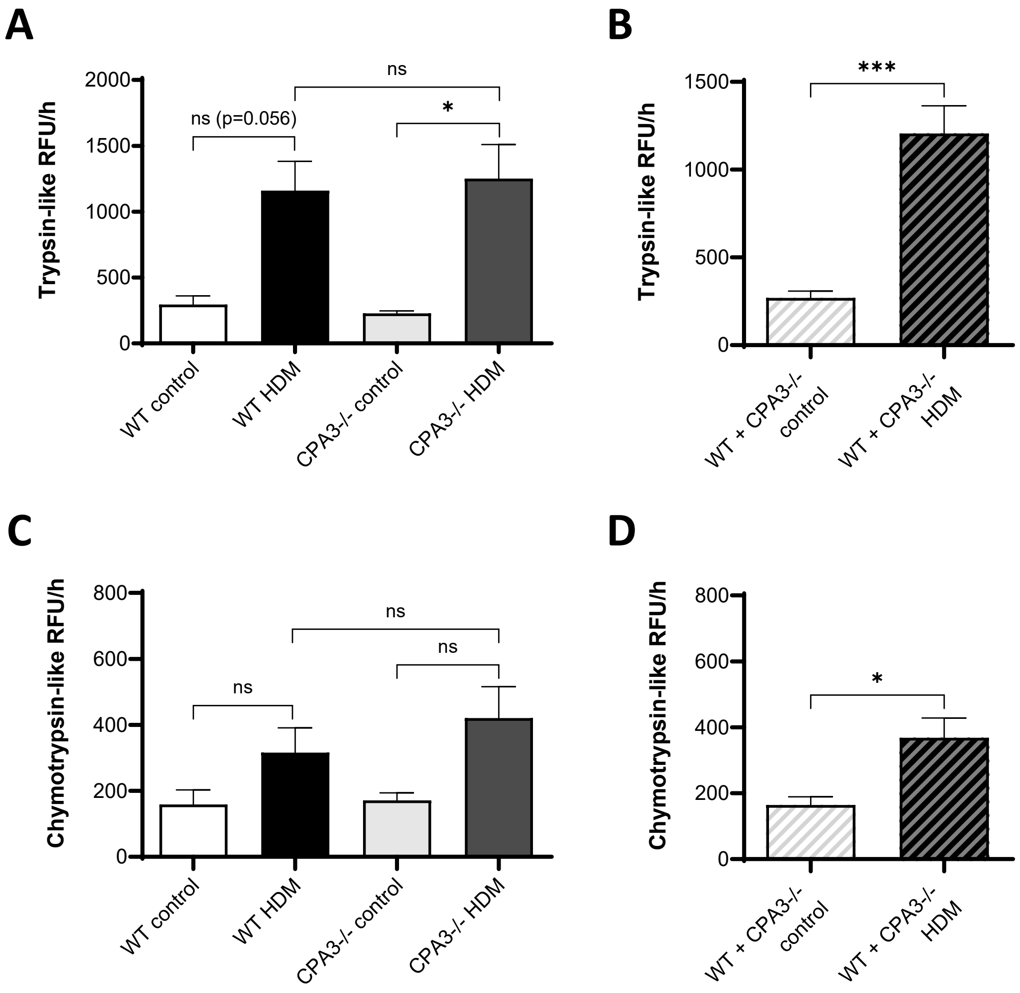

Figure S2. Increased trypsin-like and chymotrypsin-like activity in HDM-treated mice. Mice received i.n. doses of either HDM or PBS (controls) twice a week for 3 weeks. (**A**) Lung homogenates from control and HDM-treated WT and $CPA3^{-/-}$ mice were assayed for trypsin-like activity using the fluorogenic substrate Boc-Val-Pro-Arg-AMC (I-1120). (**B**) Trypsin-like activity data from WT and $CPA3^{-/-}$ mice were pooled into two groups, controls and HDM-treated. (**C**) Chymotrypsin-like activity in lung homogenates was assayed using the fluorogenic substrate Suc-Ala-Ala-Pro-Phe-AMC (I-1465). (**D**) Chymotrypsin-like activity data from WT and $CPA3^{-/-}$ mice were pooled into two groups, controls and HDM-treated. Results are expressed as mean ± SEM. *P < 0.05, **P < 0.01 or ***P < 0.001 (1-way ANOVA). (**A, C**) n = 2-4 mice per group. (**B, D**) n= 5 for controls and 8 for HDM-treated group.
